# Diagnostic performance of an algorithm for automated collateral scoring on computed tomography angiography

**DOI:** 10.1007/s00330-022-08627-4

**Published:** 2022-03-04

**Authors:** Lennard Wolff, Simone M. Uniken Venema, Sven P. R. Luijten, Jeannette Hofmeijer, Jasper M. Martens, Marie Louise E. Bernsen, Adriaan C. G. M. van Es, Pieter Jan van Doormaal, Diederik W. J. Dippel, Wim van Zwam, Theo van Walsum, Aad van der Lugt, on behalf of the MR CLEAN Registry investigators

**Affiliations:** 1 Department of Radiology and Nuclear Medicine, Erasmus MC University Medical Center, Rotterdam, The Netherlands; 2 Department of Neurology and Neurosurgery, University Medical Center Utrecht, Utrecht, The Netherlands; 3 Department of Neurology, Rijnstate Hospital, Arnhem, The Netherlands; 4 Department of Radiology, Rijnstate Hospital, Arnhem, The Netherlands; 5 Department of Radiology, Leiden University Medical Center, Leiden, The Netherlands; 6 Department of Neurology, Erasmus MC University Medical Center, Rotterdam, The Netherlands; 7 Department of Radiology and Nuclear Medicine, Maastricht University Medical Center, Maastricht, The Netherlands

**Keywords:** Ischemic stroke, Collateral circulation, Algorithms

## Abstract

**Objectives:**

Outcome of endovascular treatment in acute ischemic stroke patients depends on collateral circulation to provide blood supply to the ischemic territory. We evaluated the performance of a commercially available algorithm for assessing the collateral score (CS) in acute ischemic stroke patients.

**Methods:**

Retrospectively, baseline CTA scans (≤ 3-mm slice thickness) with an intracranial carotid artery (ICA), middle cerebral artery segment M1 or M2 occlusion, from the MR CLEAN Registry (*n* = 1627) were evaluated. All CTA scans were evaluated for visual CS (0–3) by eight expert radiologists (reference standard). A Web-based AI algorithm quantified the collateral circulation (0–100%) for correctly detected occlusion sides. Agreement between visual CS and categorized automated CS (0: 0%, 1: > 0– ≤ 50%, 2: > 50– < 100%, 3: 100%) was assessed. Area under the curve (AUC) values for classifying patients in having good (CS: 2–3) versus poor (CS: 0–1) collaterals and for predicting functional independence (90-day modified Rankin Scale 0–2) were computed. Influence of CTA acquisition timing after contrast material administration was reported.

**Results:**

In the analyzed scans (*n* = 1024), 59% agreement was found between visual CS and automated CS. An AUC of 0.87 (95% CI: 0.85–0.90) was found for discriminating good versus poor CS. Timing of CTA acquisition did not influence discriminatory performance. AUC for predicting functional independence was 0.66 (95% CI 0.62–0.69) for automated CS, similar to visual CS 0.64 (95% CI 0.61–0.68).

**Conclusions:**

The automated CS performs similar to radiologists in determining a good versus poor collateral score and predicting functional independence in acute ischemic stroke patients with a large vessel occlusion.

**Key Points:**

• *Software for automated quantification of intracerebral collateral circulation on computed tomography angiography performs similar to expert radiologists in determining a good versus poor collateral score.*

• *Software for automated quantification of intracerebral collateral circulation on computed tomography angiography performs similar to expert radiologists in predicting functional independence in acute ischemic stroke patients with a large vessel occlusion.*

• *The timing of computed tomography angiography acquisition after contrast material administration did not influence the performance of automated quantification of the collateral status.*

**Supplementary Information:**

The online version contains supplementary material available at 10.1007/s00330-022-08627-4.

## Introduction

Endovascular treatment (EVT) is the preferred option for patients with ischemic stroke due to large intracranial vessel occlusion [1]. Clinical outcome after EVT is affected by patient characteristics, workflow parameters, and imaging-derived parameters [2]. An important imaging-based parameter which predicts outcome is the extent of collateral circulation to provide blood supply to the ischemic territory [3]. This can be used to select patients who may benefit from endovascular treatment [4]. Conventional collateral scoring systems use a categorical scale, which is based on assessing visual differences in vascular filling of the middle cerebral arteries (MCA) regions between the affected and unaffected hemispheres [5]. However, visual collateral scores (vCS) assessed by a radiologist or stroke physician have substantial inter- and intra-observer variations [6].

Fast, reliable, and consistent automated scoring of the collaterals could not only reduce time delay before initiating treatment but also reduce human error in judgment, which may improve treatment outcome. Moreover, since the qCS is a quantitative measure, it might give more information on the degree of collateral circulation than the visual categorical collateral score. Several automated image algorithms to assess collateral status in acute ischemic stroke patients have been developed [7–11]. The aim of this study was to evaluate the performance of a commercially available algorithm for assessing the collateral score in baseline CTA images of acute ischemic stroke patients.

## Methods

### Study population

We used data from the Multicenter Randomized Clinical Trial of Endovascular Treatment of Acute Ischemic Stroke Registry (MR CLEAN Registry) in The Netherlands, which collects consecutive patient data from all stroke intervention centres that perform endovascular treatment (EVT) [12]. Collateral score or the severity and the extent of early ischemic changes were not used as exclusion criteria for EVT. Detailed MR CLEAN Registry eligibility criteria were published previously [12].

**Fig. 1:**
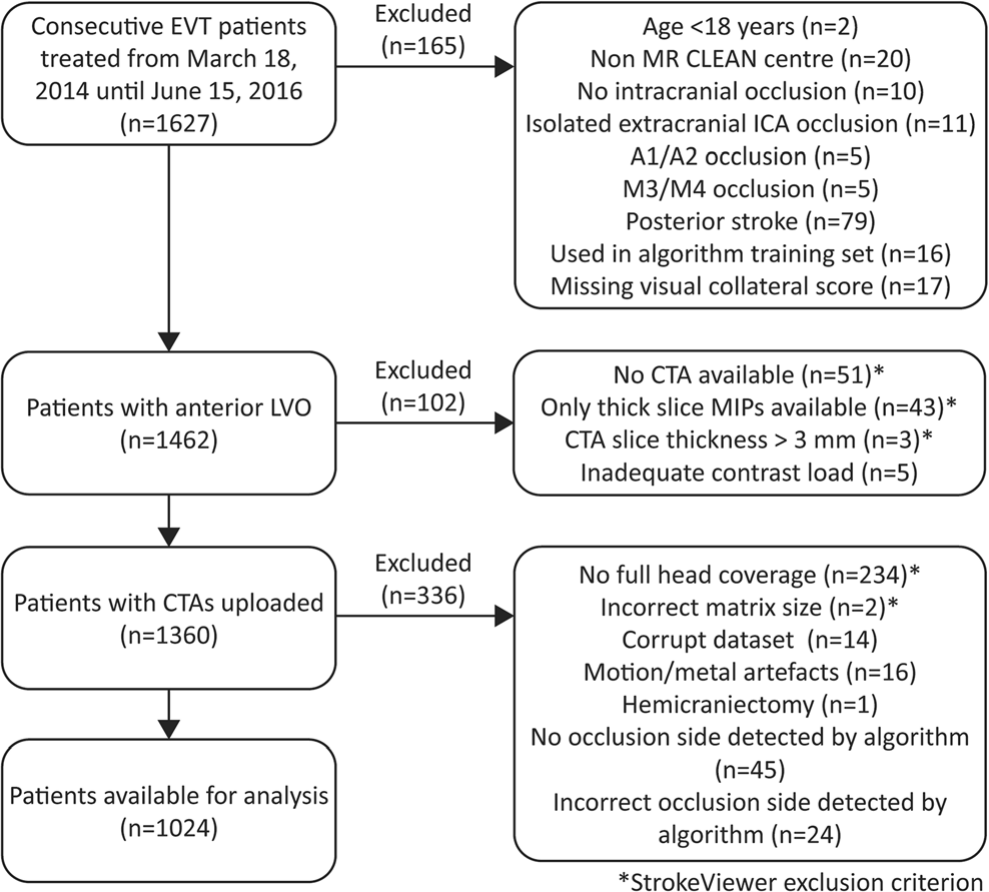
Patient selection. *StrokeViewer exclusion criterion. EVT, endovascular treatment; CTA, computed tomography angiography; LVO, large vessel occlusion; ICA, internal carotid artery; ICA, internal carotid artery terminus; M1, M1 segment of the middle cerebral artery; M2, M2 segment of the middle cerebral artery; A1/A2, A1 or A2 segment of the anterior cerebral artery; M3/M4, M3 or M4 segment of the middle cerebral artery; MIP, maximum intensity projection; mm, millimeters

The study protocol was evaluated by the Erasmus Medical Center ethics committee (Rotterdam, the Netherlands), who granted permission to carry out the study as a registry (MEC-2014-235). We selected all MR CLEAN Registry patients with a large vessel occlusion of the anterior circulation undergoing EVT from March 16, 2014, until June 15, 2016 (*n*= 1627). All patients with available baseline source CTA data were eligible for inclusion. Imaging parameters which needed to be met to process the CTA scan by StrokeViewer included axial series with a slice thickness ≤ 3 mm; slice increment equal to or smaller than slice thickness; minimum matrix size of 512 × 512; full head coverage; detection of affected cerebral hemisphere by the algorithm. Additional inclusion and exclusion criteria are shown in Fig. 1. This study was performed in accordance with the STARD guidelines for reporting diagnostic accuracy [13].

### Imaging core lab assessments

**Fig. 2:**
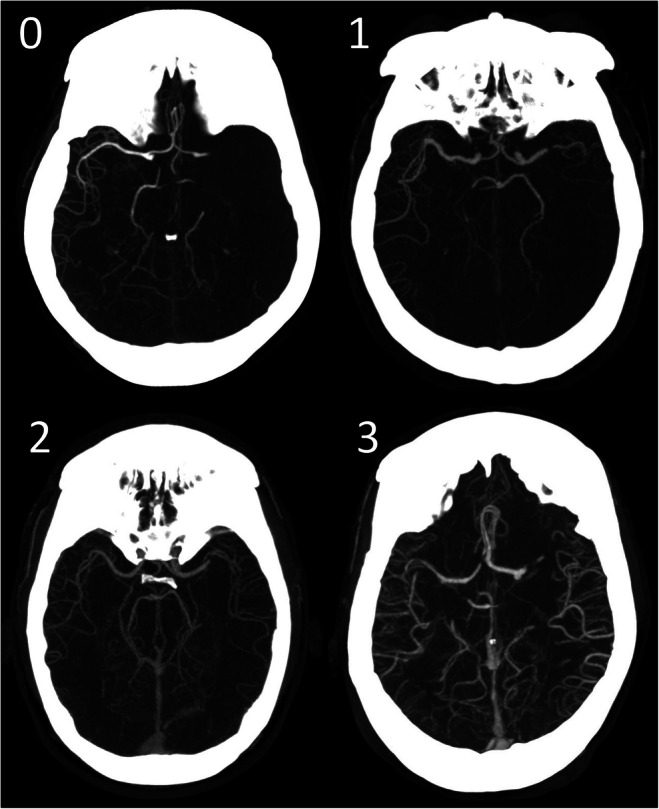
Visual collateral score grading in patients with an M1 occlusion. 0: absent collaterals, 0% filling of the occluded territory. 1: poor collaterals, > 0% and ≤ 5 0% filling of the occluded territory. 2: moderate collaterals, > 50% and < 100% filling of the occluded territory. 3: good collaterals, 100% filling of the occluded territory

All baseline CTA scans were evaluated for a 4-point collateral score by a core lab of eight expert radiologists (5 – 20 years of experience). The collateral score indicates the percentage of collateral supply in the occluded vessel territory in comparison to the contralateral side (0: absent, 1: > 0% and ≤ 50%, 2: > 50% and < 100%, 3: 100%), as shown in Fig. 2 [5]. Occlusion location was also assessed and subdivided in ICA, and M1 and M2 segments. The expert readers were blinded for automated collateral score and all clinical information except for symptomatic cerebral hemisphere.

### Automated collateral score workflow

StrokeViewer (v2.1.22, Nicolab B.V.) uses a Web-based AI algorithm based on a deep learning convolutional neural network to automatically identify large vessel occlusion location combined with automated vessel segmentations to produce automated collateral scores. StrokeViewer is hosted on the Google Cloud Platform (Google LLC). A previously published quantitative method for collateral scoring has been used as basis for the development of the current algorithm [14]. First, the DICOM requirements are checked (e.g., image modality, slice thickness, image dimensions). Second, all CTA scan slices without brain tissue are removed. Then, a brain segmentation algorithm is applied to create a brain mask. The resulting image is aligned to a reference brain atlas.

To compute a collateral score, first, the hemisphere with ischemic stroke is identified. StrokeViewer includes an occlusion detection algorithm for LVO in the anterior circulation. The occlusion location is indicated with a cube on the processed CTA scan. The occlusion detection algorithm performance is described elsewhere [15]. Once the occlusion location is detected, the collateral score is computed (quantitative scale, 0–100%) for both the specific area affected by the occlusion, defined as the area with detected vessels distal from the found occlusion, and for the whole MCA region. A score of 0% corresponds to no vascular filling in the assessed region, whereas 100% reflects non-impaired vascular filling in the assessed region. CTA scan phase (early arterial, peak arterial, equilibrium, early venous, late venous) was also detected automatically by StrokeViewer to determine the reliability of the collateral score [16].

A visual presentation of the occlusion location and the MCA vessel trees segmentation is generated and stored in DICOM format, which is accessible for the clinician. In addition, PDF reports with CTA maximum intensity projection (MIP) snapshots showing the occlusion location and MCA vessel tree segmentations, the collateral score, and other findings are generated. StrokeViewer produced results but showed a warning when scan quality deemed to be insufficient (early arterial, late venous or unknown scan phase, slice thickness > 2.0 mm). All information is accessible in StrokeViewer. The clinician is notified by email once the results are generated. Since we did not develop StrokeViewer, more algorithm details were not available to us.

### Automated collateral score assessments

CTA scan processing time was defined as the time between the finished CTA series upload and the receipt of a notification that the results are generated. StrokeViewer was used to access the generated PDF reports with the algorithm results for each patient. The results were subsequently stored in a database for further analysis.

### Statistical analysis

All scans with a visual and automated collateral score were analyzed. Analysis was done with the quantitative automated collateral score (qCS) for the affected region, unless stated otherwise. From all scans included in the analyses, a random sample of 200 CTA scans was scored by two expert radiologists to provide interobserver agreements as percentage and as intraclass correlation coefficient (ICC) using a two-way random-effects, absolute agreement, single-rater/measurement model (ICC [2,1]). We presented the distribution of qCS per vCS (0–3). To assess collateral score (CS) agreement between the software and expert readers, the qCS was categorized in 4 groups (0: 0%, 1: > 0–≤ 50%, 2: > 50–≤ 100, 3: 100%). Agreement between the qCS by the algorithm and the vCS by expert readers was calculated for all CS categories (0–3) and for dichotomized vCS categories (poor: 0–1, good: 2–3). Results were displayed in an error matrix. Scans with a difference > 1 between the vCS and categorized qCS were reviewed and rescored by the first and last author independently (L.W., A.L.) and findings were reported. Agreement was also assessed with the ICC using a one-way random-effects, absolute agreement, single-rater/measurement model (ICC [1,1]). The strictest ICC model was used because a selection of readers out of a panel of multiple expert readers assessed the collateral score [17]. For the ICC, values less than 0.5 are indicative of poor reliability, values between 0.5 and 0.75 indicate moderate reliability, values between 0.75 and 0.9 indicate good reliability, and values greater than 0.90 indicate excellent reliability.

The dichotomized vCS was used to calculate the area under receiver operating characteristic curves (AUC) for the qCS of the affected region and the qCS of the whole MCA region for all occlusion locations combined and for the different occlusion locations (ICA, M1 and M2 segments). Next, qCS performance was computed for sufficient-quality scans and insufficient-quality scan categories (according to StrokeViewer) separately.

Finally, AUCs were computed for predicting functional independence (90-day modified Rankin Scale 0–2) with vCS (0–3), categorized qCS (0–3), and qCS (continuous) of the affected region and qCS (continuous) of the whole MCA region. Differences in AUC were evaluated using the method of DeLong [18]. When appropriate, 95% confidence intervals (CI) were reported. Statistical analyses were performed using the SPSS software package (version 25.0.0.1) and R statistical software (version 4.0.4) with R package pROC.

## Results

Data from 1024 patients were available for analysis (Fig. 1). CTA scans were acquired using 28 different scanner types from all major vendors (Philips, Siemens, Toshiba, Canon Medical, GE Healthcare) in > 50 hospitals. Mean processing time from finished upload to qCS result was 5 min (SD ± 72 seconds). Baseline characteristics are reported in the supplementary materials, table 1.

**Fig. 3:**
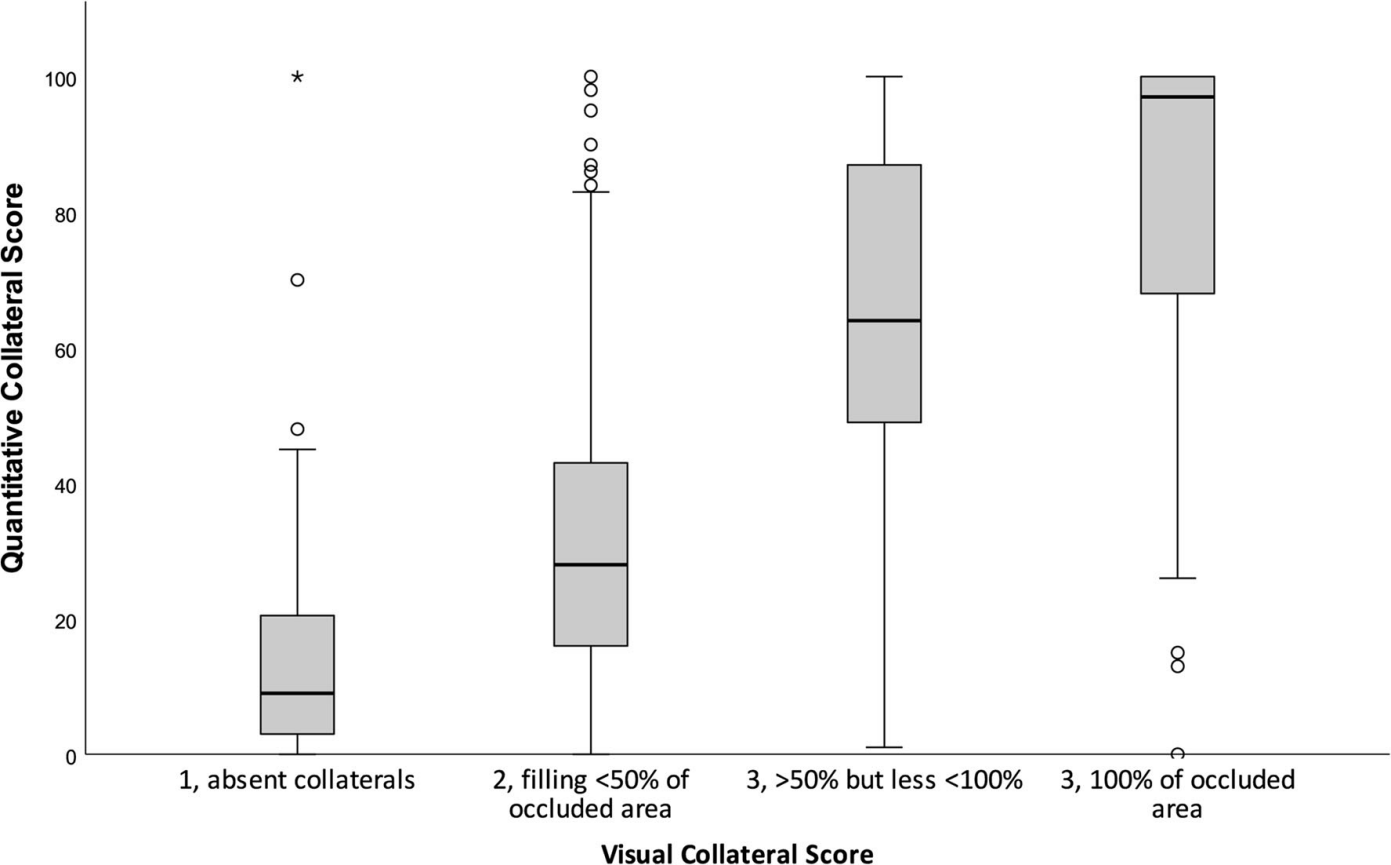
Quantitative collateral score distribution for visual collateral score categories. Distribution of quantitative collateral scores per visual collateral score. Box plots with median, interquartile range, and 95% confidence intervals. Circle: one patient. Asterisk: four patients

For the vCS, we found an interobserver agreement of 65% and an ICC of 0.66 (95% CI: 0.55–0.75) between two expert radiologists in a random sample of 200 analyzed CTA scans. A review of the disconcordant cases with a difference of > 1 between the vCS and categorized qCS (*n* = 37) showed a difference of 0 (*n* = 22) or 1 (*n* = 9) with qCS, or an incorrect vessel segmentation by StrokeViewer (*n* = 6). The distribution of qCS per vCS (0–3) is displayed in a boxplot (Fig. 3). After categorizing the qCS in collateral score 0–3 and comparing it to the visual CS, we found 59% agreement, 37% disagreement with a score difference of 1, 3% disagreement with a score difference of 2, and 1% disagreement with a score difference of 3 (Table 1). CS dichotomization (poor: 0–1, good: 2–3) resulted in an agreement of 81% (Table 1). Comparing the categorized qCS (0–4) to the visual CS resulted in a moderate ICC of 0.60 (95% CI: 0.55–0.62), which increased to 0.61 (95% CI: 0.57–0.65) after CS dichotomization.

**Table 1:**
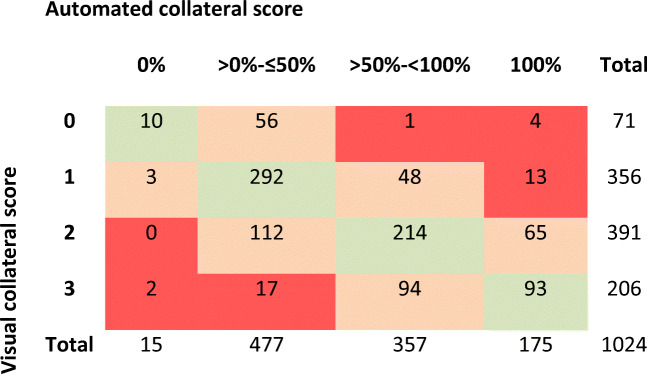
Error matrix for visual collateral score vs. automated collateral score

After dichotomizing the vCS in poor and good collaterals, overall AUC was 0.87 (95% CI: 0.85–0.90) for discrimination with automated collateral score (Table 2). When using the whole MCA region as target downstream territory, in comparison to using the region affected by the found occlusion, the performance was significantly lower (*p* < 0.01). For patients with an ICA occlusion (AUC: 0.89; 95% CI 0.85–0.93) and M1 occlusion (AUC: 0. 88; 95% CI 0.85–0.91) the performance was better than for M2 occlusions (AUC: 0.77; 95% CI 0.68–0.86) (Table 2).

**Table 2:** ROC curve characteristics for predicting dichotomized visual collateral score using the quantitative collateral score

Occlusion location (%)	*n*	Affected MCA region	Whole MCA region	AUC difference*
		AUC (95% CI)	AUC (95% CI)	*p*
All (100)	1024	0.87 (0.85–0.90)	0.85 (0.82–0.87)	< 0.01
ICA (24.9)	255	0.89 (0.85–0.93)	0.89 (0.85–0.93)	0.67
M1 (62.8)	643	0.88 (0.85–0.91)	0.84 (0.81–0.88)	< 0.01
M2 (12.3)	126	0.77 (0.68–0.86)	0.76 (0.67–0.85)	0.52

Dependent variable: dichotomized visual collateral score (0-1: poor collaterals, 2-3: good collaterals). *MCA*, middle cerebral arteries; *AUC*, Area Under the Curve; *ICA*, internal carotid artery terminus; *M1*, M1 segment of the middle cerebral artery; *M2*, M2 segment of the middle cerebral artery. *Using the Delong test

Of the analyzed patients, 40% (*n*=1024) had a CTA scan with insufficient quality according to StrokeViewer. Compared to sufficient-quality CTA scans, qCS performance was similar for all CTA scans with insufficient quality: early arterial phase scans (*n* = 246, *p* = 0.62), late venous phase scans (*n* = 72, *p* = 0.09), and scans with a slice thickness >2.0 mm (*n* = 111, *p* = 0.96). Since qCS performance was not influenced by appointed scan quality, we included and analyzed all patients (*n* = 1024) (Table 3).

**Table 3:** ROC curve characteristics for predicting dichotomized visual collateral score using the quantitative collateral score

Scan phase^a^ (%)	*n*	Occluded MCA region
		AUC (95% CI)
All (100)	1024	0.87 (0.85–0.90)
Early arterial (24.0)	246	0.86 (0.81–0.91)
Peak arterial (25.4)	260	0.92 (0.88–0.95)
Equilibrium (25.8)	264	0.88 (0.83–0.92)
Peak venous (17.7)	181	0.85 (0.79–0.91)
Late venous (7.0)	72	0.76 (0.64–0.89)
Slice thickness (%)		
> 2 mm (10.8)	111	0.87 (0.81–0.94)

Dependent variable: dichotomized visual collateral score (0-1: poor collaterals, 2-3: good collaterals). *MCA*, middle cerebral arteries; *AUC*, Area Under the Curve; *mm*, millimeters. ^a^Scan phase not detected in 1 patient

The AUC for predicting functional independence (90-day modified Rankin Scale 0–2) with the vCS was 0.64 (95% CI: 0.61–0.68) and this increased to 0.66 (95% CI 0.62–0.69) when using qCS (Table 4). The AUC remained unchanged when using the whole MCA region instead of the occluded MCA region to compute the qCS (Table 4). AUC decreased to 0.63 (95% CI 0.60–0.67) after qCS categorization in 4 groups (0: 0%, 1: > 0– ≤ 50%, 2: > 50–< 100%, 3: 100%), but in comparison to the vCS, this difference was not statistically significant (*p* = 0.29) (Table 4).

**Table 4:** Predicting functional independence (90-day mRS 0–2) with collateral score

Collateral score	AUC (95% CI)	AUC difference with visual collateral score* – *p*
Visual	0.64 (0.61–0.68)	N.A.
Automated categorized 0–3**	0.63 (0.60–0.67)	0.61
Automated continuous (affected MCA)	0.66 (0.62–0.69)	0.29
Automated continuous (whole MCA)	0.66 (0.62–0.69)	0.25

*MCA*, middle cerebral arteries; *AUC*, Area Under the Curve. Dependent variable: dichotomized visual collateral score (0–1: poor collaterals, 2–3: good collaterals). *Using the Delong test **0: 0%, 1: > 0%–≤ 50%, 2: > 50%–< 100%, 3: 100%

## Discussion

In this study, we analyzed the performance of automated collateral scoring on baseline CTA scans in 1024 acute ischemic stroke patients with a large vessel occlusion in the MCA territory.

This study demonstrated an agreement of 59% between the categorized automated collateral score and the visual collateral score, which increased to 81% after classifying patients in having good (CS: 2–3) or poor (CS: 0–1) collaterals. This is comparable to interrater agreements in this study and previous studies [4, 6, 8]. The qCS performance for M2 occlusions was lower than that for M1 and ICA, which could be explained by misclassifications of M2 occlusions by the thrombus detection algorithm [15]. The timing of CTA acquisition after administering intravenous contrast did not influence quantitative CS performance. The accuracy in predicting functional independence (90-day mRS 0–2) was similar for automated and visual collateral scoring.

Several studies have been published on automation of the CS with comparable performance when compared to visual scoring. A previous study on 442 patients reported the results of the same collateral score algorithm and compared it to the visual score which resulted in a Spearman *ρ* of 0.75 [9]. Another study with 89 patients from Grunwald et al assessed the performance of an automated CTA collateral score algorithm (Brainomix Ltd.), which uses a combination of classical image processing techniques and machine learning classifiers to produce a categorical CS of 0–3 [8]. The authors reported 90% agreement and an ICC of 0.93 with a visual collateral score based on consensus between 3 neuroradiologists. However, the neuroradiologists were informed on the automated CTA collateral score [8]. Recently, a study on 269 patients reported the performance of an algorithm using a deep learning convolutional neural network to produce both a categorical CS (0–3) and qCS [7]. The reference standard was produced by two radiologists, and a third radiologist in case of disagreement, who all scored the visual CS independently on baseline CTA imaging. For the categorical CS in comparison to the visual CS, an agreement of 80% was reported, which increased to 90% for the dichotomized CS (0–1, 2–3) [7]. Lastly, a study with 86 patients published results from ColorViz, a tool to assess collateral circulation during stroke [10]. Two neuroradiologists scored collateral status on multiphase CTA (mCTA) using a 6-point scale [19] which was compared to a 3-point visual scale that scored the automated color-coded summation maps displaying the intracranial vasculature using the mCTA scan. When using this tool, neuroradiological expertise is still obligatory, since no automated score is produced by ColorViz.

Although collateral status is not commonly used in clinical practice in treatment decisions, it is an established treatment effect modifier [4]. Also, collateral status predicts outcome [20]. Therefore, fast, reliable, and consistent collateral scoring is of utmost importance. The predictive value of CS is low with an AUC of 0.64 (vCS) and 0.66 (qCS). However, this is a prediction model with just one parameter. A recently published multivariable model to predict functional independence showed an AUC of 0.73–0.80 in 4 cohorts, but needed 11 baseline clinical and radiological parameters to achieve this [1]. From these parameters, collateral score was the strongest radiological predictor for outcome, even surpassing predictive performance of the occlusion location [2]. This makes the collateral score the excellent candidate for automation in order to facilitate the user with all associated benefits (consistent, always available, visualization of intracranial vessels). This study demonstrates that qCS provides a reliable replacement for visual collateral scoring. Neuroradiological expertise for reliable collateral scoring is not always available in acute stroke, especially during shift hours or in smaller hospitals. Since time is brain -every minute of untreated ischemic stroke, millions of brain cells die-, automated tools that have the potential to expedite stroke treatment may contribute to better outcome. After non-contrast CT, CTA is the most commonly requested imaging in suspected stroke patients, which makes a CTA-based qCS easy to implement. However, this study could not demonstrate a better prediction of outcome with QCS compared to visual score. This is in line with a previous study which reported similar performance of vCS versus qCS [9]. Prediction and prognostication in clinical practice should in the end be performed with a model that contains both clinical and imaging parameters [21].

There are a few limitations to our study. Due to the large sample size, vCS was scored by only one observer per scan. We addressed this issue by a blind review by the first and last authors independently of scans with vCS outliers in comparison to qCS. From the patients with anterior LVO, 26% of patients were excluded due to absence of usable CTA imaging. This was mainly due to incomplete head coverage (234/385) which could be solved in future studies by emphasizing importance of displaying the complete brain in patients with suspected ischemic stroke. This not only enables algorithms to process a larger percentage of scans, but also helps physicians minimize the risk of missing a diagnosis in the non-displayed part of the head. A total of 5% of all anterior LVO patients were excluded because no occlusion side was detected (45/69, supplementary materials, table 2) or an incorrect side was appointed as the affected hemisphere (24/69, supplementary materials, table 3). If no occlusion side was detected, qCS was not assessed, but an incorrectly detected affected hemisphere still resulted in a collateral score, which was mainly higher than the reference visual score, since qCS is computed by comparing the affected hemisphere to the unaffected hemisphere. In future versions of the automated collateral scoring algorithm, this could be addressed by retraining with more cases and focus on occlusion side, especially in cases with high CS. A more pragmatic approach would be to make it possible to give the symptomatic side as input to the algorithm. This enables the algorithm to focus on the corresponding hemisphere, which could reduce errors.

Although algorithm performance was independent of scan phase, a suboptimal scan phase could give an erroneous interpretation of the collateral score in an individual. If a CTA scan is acquired in the late venous phase, the amount of contrast visible in the healthy hemisphere could be lower in comparison to the affected hemisphere due to delayed filling of the vasculature [22]. This can result in an apparent high vCS, even when there is a clear deficit visible on CT-perfusion imaging, but delayed filling in the late venous phase is associated with poor baseline collateral status at baseline [22]. An early arterial scan phase could give similar issues due to delayed filling. This emphasizes the importance of scan phase awareness when assessing the collateral status.

The mean processing time to produce qCS was 5 min. This should be improved to be able to beat a neuroradiologist in providing a collateral score. Nevertheless, 24/7 availability of computer algorithms, the consistent qCS scoring, and the visualization of intracranial vessels are valuable characteristics for physicians treating patients with a suspected stroke.

## Conclusions

This automated collateral score deep learning–based algorithm performs similar to expert radiologists in categorizing patients with a good versus poor collateral score and in predicting functional independence in acute ischemic stroke patients with a large vessel occlusion.

## Supplementary Information

ESM 1 (DOCX 36 kb)

## Abbreviations

AUC: Area under the curve
CI: Confidence interval
CS: Collateral score
EVT: Endovascular treatment
LVO: Large vessel occlusions
MCA: Middle cerebral arteries
MIP: Maximum intensity projection
qCS: Quantitative automated collateral score
vCS: Visual collateral score

## Declarations

### Guarantor

The scientific guarantor of this publication is Aad van der Lugt.

### Statistics and biometry

No complex statistical methods were necessary for this paper.

### Study subjects or cohorts overlap

Some study subjects or cohorts have been previously reported in other MR CLEAN Registry publications.

### Methodology

• retrospective (this study)

• randomized, controlled, open-label, phase 3 trial

• multicenter study
