# Supplementary material for: Diagnostic performance of an algorithm for automated collateral scoring on computed tomography angiography
Source: Eur Radiol. 2022 Mar 4;32(8):5711–8. doi: 10.1007/s00330-022-08627-4 (PMC9279191; doi:10.1007/s00330-022-08627-4)
Supplement: Supplementary file 1 — (DOCX 36 kb) [file 330_2022_8627_MOESM1_ESM.docx]

Diagnostic performance of an algorithm for automated collateral scoring on computed tomography angiography

## supplemental materials

**Table 1.** Baseline characteristics of the 1024 analysed patients

| **Demographics** | |
| --- | --- |
| Age - year |  |
| Median | 71.2 |
| Interquartile range | 50.8–76.9 |
| Male sex – no. (%) | 543 (53.0) |
| **Medical history** | |
| Prestroke modified Rankin scale score – no. (%)^a^ |  |
| 0 | 666 (65.0) |
| 1 | 139 (13.6) |
| 2 | 83 (8.1) |
| >2 | 118 (11.5) |
| Systolic blood pressure – mm Hg^b^ | 149.2±24.9 |
| Treatment with IV alteplase – no. (%) | 777 (75.9) |
| **Imaging** | |
| Right hemisphere, n (%) | 480 (53.1) |
| Occluded segment – no./total no. (%) |  |
| ICA | 255 (24.9) |
| M1 | 643 (62.8) |
| M2 | 126 (12.3) |
| ASPECTS on NCCT, median (IQR)^c^ | 9 (7–10) |
| Visual collaterals, n (%) |  |
| Grade 0 | 71 (6.9) |
| Grade 1 | 356 (34.8) |
| Grade 2 | 391 (38.2) |
| Grade 3 | 206 (20.1) |
| Quantitative collaterals, n (%) |  |
| 0 - < 25% | 15 (1.5) |
| 25 - < 50% | 477 (46.6) |
| 50 - < 75% | 357 (34.9) |
| 75 – 100% | 175 (17.1) |

^a^Scores on the modified Rankin scale (mRS) of functional disability range from 0 (no symptoms) to 6 (death). A score of 2 or less indicates functional independence. Data on mRS at baseline were missing for 18 patients.

^b^Data on systolic blood pressure at baseline were missing for 28 patients.

^c^The Alberta Stroke Program Early Computed Tomography Score (ASPECTS) is a measure of the extent of stroke. Scores ranges from 0 to 10, with higher scores indicating fewer early ischemic changes. Scores were not available for 24 patients.

**Occlusion side detection errors**

5% of all anterior LVO patients (n=1360) were excluded because no occlusion side was detected (45/69) or an incorrect side was appointed as the affected hemisphere (24/69). Details on these cases can be found in table 1 (no occlusion side found) and table 2 (incorrect side appointed as affected hemisphere).

**Table 2.** Cases without detection of occlusion side by the algorithm.

| Case | Occlusion side | Most proximal occlusion location | Collateral score | ASPECTS | phase | slicethickness |
| --- | --- | --- | --- | --- | --- | --- |
|  | Core Lab | Core Lab | Core Lab | Core Lab | Algorithm | CTA scan |
| 1 | Right | M2 | 0 | 10 | Peak Venous | 1,3 |
| 2 | Right | M2 | 0 | 10 | Equilibrium | 2,0 |
| 3 | Left | M2 | 0 | 10 | Equilibrium | 2,0 |
| 4 | Right | ICA-T | 1 | 7 | Peak Arterial | 1,0 |
| 5 | Left | ICA-T | 1 | 6 | Early Arterial | 1,0 |
| 6 | Right | M1 | 1 | 10 | Late Venous | 1,0 |
| 7 | Right | M1 | 1 | 10 | Equilibrium | 0,6 |
| 8 | Right | M2 | 1 | 10 | Early Arterial | 2,0 |
| 9 | Right | M2 | 1 | 8 | Early Arterial | 3,0 |
| 10 | Left | Extracranial ICA (with tandem lesion) | 2 | 10 | Peak Venous | 2,0 |
| 11 | Left | ICA-T | 2 | 6 | Early Arterial | 2,0 |
| 12 | Left | ICA-T | 2 | 9 | Equilibrium | 1,0 |
| 13 | Left | ICA-T | 2 | 5 | Early Arterial | 3,0 |
| 14 | Right | Intracranial ICA | 2 | 9 | Peak Venous | 2,0 |
| 15 | Right | M1 | 2 | 5 | Peak Venous | 3,0 |
| 16 | Left | M1 | 2 | 9 | Peak Arterial | 0,9 |
| 17 | Right | M2 | 2 | 10 | Peak Arterial | 0,8 |
| 18 | Right | M2 | 2 | 5 | Peak Arterial | 0,8 |
| 19 | Right | M2 | 2 | 10 | Equilibrium | 1,3 |
| 20 | Right | M2 | 2 | 10 | Equilibrium | 2,0 |
| 21 | Right | M2 | 2 | 10 | Peak Arterial | 0,5 |
| 22 | Right | M2 | 2 | 7 | Peak Venous | 0,9 |
| 23 | Left | M2 | 2 | 5 | Peak Arterial | 0,8 |
| 24 | Left | M2 | 2 | 10 | Peak Arterial | 0,8 |
| 25 | Left | M2 | 2 | 10 | Equilibrium | 0,8 |
| 26 | Left | M2 | 2 | 10 | Early Arterial | 0,5 |
| 27 | Left | M2 | 2 | 2 | Equilibrium | 0,9 |
| 28 | Left | Extracranial ICA (with tandem lesion) | 3 | 10 | Late Venous | 2,0 |
| 29 | Left | Extracranial ICA (with tandem lesion) | 3 | - | Peak Arterial | 0,8 |
| 30 | Right | ICA-T | 3 | 9 | Late Venous | 2,0 |
| 31 | Left | Intracranial ICA | 3 | 10 | Peak Venous | 1,3 |
| 32 | Left | M1 | 3 | 9 | Peak Arterial | 1,0 |
| 33 | Right | M1 | 3 | 8 | Peak Arterial | 0,8 |
| 34 | Right | M1 | 3 | 10 | Peak Venous | 1,0 |
| 35 | Right | M1 | 3 | 10 | Equilibrium | 2,0 |
| 36 | Right | M1 | 3 | 10 | Peak Venous | 2,0 |
| 37 | Right | M1 | 3 | 9 | Peak Arterial | 0,5 |
| 38 | Right | M1 | 3 | 10 | Peak Arterial | 0,9 |
| 39 | Right | M2 | 3 | 10 | Peak Arterial | 1,0 |
| 40 | Right | M2 | 3 | 9 | Early Arterial | 0,5 |
| 41 | Left | M2 | 3 | 10 | Peak Venous | 1,3 |
| 42 | Left | M2 | 3 | 10 | Early Arterial | 2,0 |
| 43 | Left | M2 | 3 | 9 | Equilibrium | 2,0 |
| 44 | Left | M2 | 3 | 10 | Peak Arterial | 0,8 |
| 45 | Left | M2 | 3 | 10 | Peak Venous | 3,0 |

**Table 3.** Cases with an incorrect side appointed as affected hemisphere.

| Case | Occlusion side | | Most proximal occlusion location* | | Collateral score | | Quantitative collateral score | Detected acquisition phase | ASPECTS** | Slice thickness (mm) |
| --- | --- | --- | --- | --- | --- | --- | --- | --- | --- | --- |
|  | Core Lab | Algorithm | Core Lab | Algorithm | Core Lab | Algorithm | Algorithm | Algorithm | Core Lab | CTA scan |
| 1 | Left | Right | ICA-T | Intracranial ICA | 0 | 3 | 100 | Late Venous | 10 | 2,0 |
| 2 | Left | Right | M1 | Not on vessel | 1 | 2 | 62 | Early Arterial | 10 | 2,0 |
| 3 | Right | Left | ICA-T | M1 | 1 | 2 | 71 | Peak Venous | 8 | 1,0 |
| 4 | Left | Right | ICA-T | M1 | 1 | 3 | 100 | Peak Arterial | 9 | 0,9 |
| 5 | Left | Right | M1 | Not on vessel | 1 | 3 | 100 | Early Arterial | 10 | 0,9 |
| 6 | Right | Left | M1 | M1 | 1 | 3 | 100 | Early Arterial | 7 | 0,9 |
| 7 | Right | Left | M1 | M2 | 1 | 3 | 100 | Equilibrium | 10 | 3,0 |
| 8 | Right | Left | M2 | Intracranial ICA | 1 | 3 | 100 | Early Arterial | 8 | 3,0 |
| 9 | Left | Right | ICA-T | Not on vessel | 2 | 1 | 47 | Peak Venous | 8 | 1,0 |
| 10 | Left | Right | M2 | ICA-T | 2 | 2 | 58 | Equilibrium | 9 | 3,0 |
| 11 | Right | Left | M1 | M1 | 2 | 2 | 61 | Equilibrium | 10 | 3,0 |
| 12 | Left | Right | ICA-T | Not on vessel | 2 | 2 | 67 | Early Arterial | 8 | 2,0 |
| 13 | Left | Right | Intracranial ICA | Intracranial ICA | 2 | 3 | 100 | Peak Arterial | 8 | 0,6 |
| 14 | Right | Left | Intracranial ICA | M1 | 2 | 3 | 100 | Early Arterial | 8 | 1,0 |
| 15 | Left | Right | ICA-T | Not on vessel | 2 | 3 | 100 | Peak Arterial | 7 | 3,0 |
| 16 | Right | Left | ICA-T | M1 | 2 | 3 | 100 | Early Arterial | 9 | 2,0 |
| 17 | Left | Right | ICA-T | Intracranial ICA | 3 | 1 | 5 | Late Venous | 10 | 1,3 |
| 18 | Left | Right | M2 | ICA-T | 3 | 1 | 24 | Late Venous | 10 | 1,0 |
| 19 | Right | Left | M1 | M1 | 3 | 1 | 35 | Peak Venous | 10 | 1,0 |
| 20 | Right | Left | ICA-T | Not on vessel | 3 | 2 | 70 | Peak Venous | 5 | 0,8 |
| 21 | Left | Right | M2 | Not on vessel | 3 | 2 | 70 | Late Venous | 9 | 1,0 |
| 22 | Right | Left | M1 | Not on vessel | 3 | 3 | 100 | Peak Arterial | 10 | 1,0 |
| 23 | Right | Left | M1 | Not on vessel | 3 | 3 | 100 | Peak Venous | - | 0,6 |
| 24 | Right | Left | M2 | Not on vessel | 3 | 3 | 100 | Late Venous | 7 | 2,0 |

*For the algorithm: a visual box was placed around the detected occlusion by the algorithm. The most proximal occlusion location within the placed box was noted by a reader (S.L.).

**The Alberta stroke programme early CT score.
